# Polymorphisms of ADME-related genes and their implications for drug safety and efficacy in Amazonian Amerindians

**DOI:** 10.1038/s41598-019-43610-y

**Published:** 2019-05-10

**Authors:** Juliana Carla Gomes Rodrigues, Marianne Rodrigues Fernandes, João Farias Guerreiro, Artur Luiz da Costa da Silva, Ândrea Ribeiro-dos-Santos, Sidney Santos, Ney Pereira Carneiro dos Santos

**Affiliations:** 1 Research Center of Oncology, Federal University of Pará, Belém, Brazil; 2 Laboratory of Human and Medical Genetics, Institute of Biological Science, Federal University of Pará, Belém, Brazil; 3 Genomics and Bioinformatics Laboratory, Institute of Biological Science, Federal University of Pará, Belém, Brazil

**Keywords:** Genetic markers, Population genetics

## Abstract

The variation in the allelic frequencies of polymorphic pharmacogenes among different ethnic groups may be responsible for severe adverse reactions to or altered efficacy of a wide variety of drugs. Amazonian Amerindian populations have a unique genetic profile that may have a fundamental on the efficacy and safety of certain drugs. The genetic characteristics of these populations are poorly known, which can negatively impact the systematic application of treatments guided by pharmacogenomic guidelines. We investigated the diversity of 32 polymorphisms in genes responsible for drug Absorption, Distribution, Metabolism and Excretion (ADME) in Amazonian Amerindians, and compared the findings with populations from other continents available in the 1000 Genomes database. We found significantly different (*P* ≤ 1.56E-03) allelic frequencies and genotype distributions in many study markers in comparison with African, European, American and Asian populations. Based on FST values, the Amerindian population was also the most distinct (mean FST = 0.09917). These data highlight the unique genetic profile of the indigenous population from the Brazilian Amazon region, which is potentially important from a pharmacogenetic viewpoint. Understanding the diversity of ADME- related genetic markers is crucial to the implementation of individualized pharmacogenomic treatment protocols in Amerindian populations, as well as populations with a high degree of admixture with this ethnic group, such as the general Brazilian population.

## Introduction

Inter-individual variability plays a fundamental role in the tolerance of and response to a number of different treatments that are widely used in clinical practice^1^. Much of this inter-individual variability is related to polymorphisms in the genes involved in the Absorption, Distribution, Metabolism, and Excretion (the ADME process) of drugs or the mechanisms that determine their action in the body^2,3^.

The discovery that the response to and tolerance of drugs may vary not only among individuals, but systematically among populations, in particular among different ethnic groups, had a fundamental impact on pharmacogenetic studies, leading to the development of the field of Pharmacoethnicity^4–6^. The principal determinant of this interethnic variability is the allelic frequencies of the polymorphisms found in pharmacogenes, which may vary considerably among different ethnic groups^7–9^.

The Brazilian population is characterized by its high degree of ethnic admixture^10^, being formed by three principal ancestral groups: European settlers, African slaves, and Amerindians (Native Americans). Five centuries of miscegenation of these groups have resulted in an extremely heterogeneous genetic makeup, and a considerable challenge for pharmacogenetic studies in Brazil^11–13^.

According to the most recent census (2010) of the Brazilian Institute of Geography and Statistics^14^ the Amerindian population of Brazil numbers 896 917, representing 0.47% of the country’s population. While this population is genetically distinct, few data are available on the incorporation of modern habits (industrialized foodstuffs, synthetic drugs, alcoholic beverages, etc.) into its traditional lifestyles. It nevertheless seems likely that distinct patterns of toxicity and response to pharmacological therapies will be found in this ethnic group in comparison with other populations^15,16^. However, genomic studies of pharmacogenetic biomarkers are extremely rare in Amazonian Amerindian populations.

The general lack of pharmacogenomic data for these Amerindian populations represents a major obstacle to the incorporation of Pharmacogenomics (PGx) into the development of individualized treatment protocols designed to maximize the efficacy of therapies in this ethnic group. In this context, the identification of genetic polymorphisms and molecular markers of clinical significance in these populations will be essential for the development of effective treatment protocols not only for this ethnic group, but also for the populations that are admixed with this group, such as the Brazilian one^17^.

In this context, the present study investigated a set of 32 molecular ADME markers in a combined Amazonian Amerindian population, and compared the results with populations representing five continents, obtained from the 1000 Genomes database.

## Material and Methods

### Study Populations

The study population was composed of 146 healthy individuals from three Amerindian groups located in the Amazon region of Brazil, selected from a sample database of an epidemiological study of indigenous populations of Pará State. This group included 61 individuals from the Asurini do Trocará group, 25 from the Asurini do Koatinemo group, and 60 from the Kayapó-Xicrin group. The study was approved by the National Committee for Ethics in Research (CONEP), with CAAE number 20654313.6.0000.5172. The informed consent was obtained from each study participant and all research methods in this study were performed in accordance with the approved guidelines.

For the analyses, these three populations were combined in a single group, denominated IND. For comparison with populations from other continents, we used data obtained from the 1000 Genomes, phase 3 release (available at http://www.1000genomes.org), composed of 661 individuals from Africa (AFR), 503

from Europe (EUR), 347 from the Americas (AMR), 504 from East Asia (EAS), and 489 from South Asia (SAS).

### Selection of Markers

Thirty-two polymorphisms of 16 pharmacogenes were selected based on three main criteria: (1) the marker must be involved in any of the steps of the ADME process; (2) the PGx biomarker should also have high-level clinical annotations or related VIPs in the public database of the PharmGKB (www.pharmgkb.org), or it has been pointed in the specific literature as an important biomarker (www.ncbi.nlm.nih.gov/pubmed), and (3) it should also be among the PGx biomarkers recommended for drug dosage adjustment by global drug regulatory agencies.

After all the three criteria were applied, we have selected 11 membrane transporter gene polymorphisms, 11 polymorphisms of phase I metabolizing genes, and 10 polymorphisms of other genes that are indirectly involved in the ADME process.

### Genotyping and Quality Control

The DNA was extracted from the peripheral blood of the 146 study subjects using the commercial Biopur Mini Spin Plus–250 kit (Biopur, Brazil), according to the manufacture’s recommendations. The concentration and purity of the DNA were measured with a NanoDrop 1000 spectrophotometer (Termo Fisher Scientific, Wilmington, DE).

The polymorphisms of the ADME-related genes were genotyped by allelic discrimination using the TaqMan OpenArray Genotyping technology, with a set of 32 customized assays, which were run in a QuantStudio™ 12 K Flex Real-Time PCR system (Applied Biosystems, Life Technologies, Carlsbad, USA), according to the manufacturer’s protocol. The Taqman Genotyper software was used for data analysis and to verify the accuracy of the genotype readings, as well as the quality of the genotyping. The data were then filtered for SNPs that deviated from Hardy–Weinberg equilibrium (HWE).

### Statistical analysis

The allele frequencies of the Amerindian population were obtained directly by gene counting, and compared with the other study populations (AFR, EUR, AMR, EAS, and SAS). The distribution of the genotypes among the six populations was analyzed using Fisher’s exact test. Pairwise comparisons between populations were also based on Fisher’s exact test, with the p-value being adjusted by the Bonferroni correction, with a critical p-value of 1.56E-03 being considered in all cases.

The inter-population variability of the ADME SNPs was assessed using Wright’s fixation index (F_ST_). We ran a multidimensional scaling analysis of the F_ST_ values to provide a graphical representation of the genetic differentiation of the Amerindian population in comparison with each of the five study populations. The analyses were run in SPSS v.12.0 (SPSS Inc., Chicago, IL) and Arlequin v.3.5^18^.

## Results

The three Amazonian Amerindian populations were analyzed together (IND) for the comparisons of allele and genotype frequencies, and the F_ST_ values with the five populations from the 1000 Genomes Project database (AFR, AMR, EAS, EUR, and SAS). The allele frequencies were determined for a total of 32 markers (Table 1). The Hardy-Weinberg Equilibrium (HWE) was calculated and p-values of less than 1.56E-03 were considered significant. Six of the 32 markers investigated in the Amerindian population were not in HWE – rs9524885 of the *ABCC4* gene, rs8192726 of the *CYP2A6* gene, rs1801265 and rs67376798 of the *DPYD* gene, rs3758149 of the *GGH* gene, and rs1042927 of the *RRM1* gene. These markers were thus excluded from the remaining statistical analyses.

**Table 1:** Hardy Weinberg Equilibrium and Comparison of allelic frequencies of ADME-related SNPs in Amerindian population (IND) and others continental populations (AFR, AMR, EAS, EUR, SAS).

No.	Reference SNP ID	Gene Definig SNP	ALL^a^	AFR	Allele Frequencies		EUR	SAS	Hardy-Weinberg Equilibrium (P-Value)	
					AMR	EAS			IND	
1	rs1045642	***ABCB1***	0,40	0.15	0.43	0.4	0.52	0.57	0,50	0.229
2	rs1128503	***ABCB1***	0,42	0.14	0.4	0.63	0.42	0.59	0,51	1.000
3	rs717620	***ABCC2***	0,13	0.03	0.17	0.22	0.21	0.1	0,11	0.003
4	rs4148551	***ABCC4***	0,49	0.59	0.43	0.5	0.38	0.5	0,54	0.175
5	rs3742106	***ABCC4***	0,41	0.31	0.41	0.5	0.38	0.5	0,52	0.437
6	rs9524885	***ABCC4***	0,41	0.63	0.29	0.42	0.27	0.36	0,2	**4.80E‐04**
7	rs2231142	***ABCG2***	0,12	0.01	0.14	0.29	0.09	0.1	0,43	0.475
8	rs28399433	***CYP2A6***	0,13	0.08	0.1	0.24	0.07	0.15	0,46	0.600
9	rs8192726	***CYP2A6***	0,9	0.92	0.96	0.82	0.93	0.87	0,74	**0.00E-00**
10	rs17116806	***DPYD***	0,17	0.1	0.26	0.27	0.19	0.08	0,52	0.684
11	rs1760217	***DPYD***	0,2	0.19	18	0.3	0.18	0.15	0,19	1.000
12	rs1801159	***DPYD***	0,18	0.15	0.27	0.27	0.19	0.08	0,45	0.714
13	rs1801265	***DPYD***	0,74	0.56	0.78	0.91	0.79	0.73	0,79	**1.33E-03**
14	rs3918290	***DPYD***	0	0	0	—	0	0.01	0,01	1.000
15	rs4970722	***DPYD***	0,22	0.29	0.22	0.09	0.21	0.26	0,45	0.013
16	rs55886062	***DPYD***	0	—	—	—	0	—	0,02	1.000
17	rs67376798	***DPYD***	0	0	0	—	0.01	0	0,04	**3.60E-04**
18	rs17376848	***DPYD***	0,05	0.01	0.08	0.11	0.04	0.03	0,27	0.251
19	rs4451422	***FPGS***	0,54	0.6	0.54	0.31	0.61	0.6	0,58	0.152
20	rs3758149	***GGH***	0,23	0.17	0.23	0.22	0.28	0.29	0,60	**1.6E-04**
21	rs10049380	***ITGB5***	0,66	0.54	0.61	0.68	0.81	0.68	0,30	0.217
22	rs1801131	***MTHFR***	0,25	0.15	0.15	0.22	0.31	0.42	0,09	0.002
23	rs1801133	***MTHFR***	0,25	0.09	0.47	0.3	0.36	0.12	0,23	0.006
24	rs1042927	***RRM1***	0,85	0.8	0.83	0.76	0.93	0.93	0,80	**1.37E-03**
25	rs12806698	***RRM1***	0,23	0.03	0.22	0.3	0.28	0.36	0,42	0.327
26	rs2270860	***SLC22A7***	0,46	0.72	0.38	0.37	0.34	0.4	0,43	0.850
27	rs4149178	***SLC22A7***	0,19	0.34	0.21	0.05	0.17	0.13	0,36	0.202
28	rs747199	***SLC29A1***	0,15	0.01	0.18	0.27	0.22	0.1	0,64	0.006
29	rs760370	***SLC29A1***	0,3	0.26	0.37	0.3	0.4	0.2	0,40	0.690
30	rs1042522	***TP53***	0,54	0.33	0.68	0.59	0.71	0.51	0,73	0.073
31	rs11479	***TYMP***	0,14	0.03	0.17	0.29	0.07	0.19	0,26	0.453
32	rs1801019	***UMPS***	0,19	0.15	0.26	0.17	0.15	0.23	0,54	0.324

^a^ALL represents the mean values of the five continental populations (AFR, AMR, EAS, EUR and SAS).

**Figure 1:**
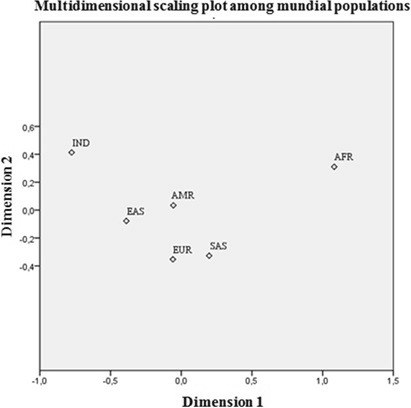
Multidimensional scaling plot illustrating the grouping of ethnic populations according to the genetic profile of the 26 ADME-markers.

The multidimensional scaling (MDS) analysis of the F_ST_ values for each pairwise comparison revealed the similarities and differences in the 26 SNPs related to the ADME drug process (Fig. 1; Table 2). The graph highlights population clusters, which indicate a very distinct genetic profile between the Amerindian and African populations, which are isolated at the extremities of the plot. The genetic profile of the Amerindian population is most similar to those of the American and East Asian populations.

**Table 2:** Pairwise FST among Amerindians and the five continental populations from 1000 genomes database.

	AFR	AMR	EAS	EUR	IND	SAS
AFR	—					
AMR	0.09588	—				
EAS	0.13062	0.03255	—			
EUR	0.11534	0.01477	0.04566	—		
IND	0.18313	0.06042	0.05831	0.09851	—	
SAS	0.10250	0.04414	0.04690	0.03268	0.09548	—

The 26 SNPs were also analyzed in pairwise comparisons between the IND population and the mean values for the five 1000 Genomes populations (ALL), as well as each of these populations individually, in order to characterize the unique aspects of the genetic profile of the Amerindian population, in comparison with those of the other populations (Table 3).

**Table 3:** Pairwise comparison of genotypic frequencies in Amerindians with each one of the five continental populations from 1000 genomes database.

No.	Reference SNP ID	Gene Defining SNP	IND versus ALL	IND versus AFR	Pairwise Comparison (P-Value)		IND versus EUR	IND versus SAS
					IND versus AMR	IND versus EAS		
1	rs1045642	***ABCB1***	**0,000351**	**8,569E-034**	0,087509	0,004795	0,459556	**0,024838**
2	rs1128503	***ABCB1***	0,010039	**5,748E-039**	0,012830	0,003571	0,459556	0,082449
3	rs717620	***ABCC2***	0,029090	**3,565E-007**	0,001932	**0,000087**	**0,000010**	0,059195
4	rs4148551	***ABCC4***	0,217774	0,117681	0,012613	0,375018	**0,000090**	0,177281
5	rs3742106	***ABCC4***	0,004675	**4,441E-009**	0,014532	0,732544	**0,000521**	0,468344
6	rs2231142	***ABCG2***	**2,336E-044**	**1,045E-085**	**7,047E-019**	**0,000050**	**4,795E-032**	**3,619E-033**
7	rs28399433	***CYP2A6***	**3,368E-042**	**7,608E-048**	**4,113E-031**	**1,402E-011**	**1,901E-046**	**1,975E-022**
8	rs17116806	***DPYD***	**1,669E-033**	**6,134E-046**	**5,005E-010**	**2,948E-010**	**1,522E-019**	**1,035E-043**
9	rs1760217	***DPYD***	0,833815	0,995618	0,953805	0,004403	0,912777	0,395147
10	rs1801159	***DPYD***	**2,491E-021**	**8,065E-024**	**0,000002**	**3,785E-007**	**2,730E-015**	**1,776E-038**
11	rs3918290	***DPYD***	0,002800	0,003626	0,024202	0,002015	0,103855	0,328929
12	rs4970722	***DPYD***	**5,305E-017**	**2,348E-007**	**3,392E-012**	**1,015E-041**	**2,247E-014**	**1,952E-009**
13	rs55886062	***DPYD***	0,000002	**0,000149**	0,001817	**0,000446**	0,002224	**0,000502**
14	rs17376848	***DPYD***	**3,229E-043**	**2,087E-048**	**9,420E-012**	**3,681E-009**	**4,826E-027**	**1,008E-026**
15	rs4451422	***FPGS***	0,379278	0,815190	0,347094	**4,991E-015**	0,230975	0,256130
16	rs10049380	***ITGB5***	**1,651E-026**	**1,252E-009**	**1,307E-012**	**7,000E-024**	**4,073E-051**	**7,416E-021**
17	rs1801131	***MTHFR***	**1,016E-009**	0,001766	0,001990	**1,000E-007**	**4,887E-014**	**4,301E-025**
18	rs1801133	***MTHFR***	0,299989	**3,303E-010**	**3,500E-014**	0,021084	**0,000004**	**1,033E-007**
19	rs12806698	***RRM1***	**5,917E-010**	**4,437E-059**	**2,146E-008**	0,001617	**0,000164**	0,069652
20	rs2270860	***SLC22A7***	0,495893	**2,488E-017**	0,355337	**0,180118**	0,042493	0,563101
21	rs4149178	***SLC22A7***	**3,976E-008**	0,593274	**0,000123**	**1,956E-032**	**1,975E-009**	**9,646E-015**
22	rs747199	***SLC29A1***	**1,111E-020**	**1,000E-069**	**9,135E-009**	0,000141	**7,604E-007**	**1,067E-018**
23	rs760370	***SLC29A1***	0,011188	**0,000199**	0,799858	0,012188	0,965590	**8,367E-009**
24	rs1042522	***TP53***	**4,888E-007**	**4,528E-029**	0,103918	**0,000133**	0,115445	**6,061E-009**
25	rs11479	***TYMP***	0,000019	**6,933E-032**	0,011643	0,582035	**4,960E-014**	0,025776
26	rs1801019	***UMPS***	**5,623E-040**	**1,341E-037**	**5,362E-012**	**5,505E-026**	**1,492E-034**	**8,120E-016**

In the comparison between the IND and the AFR populations, the distribution of 20 polymorphisms of 14 genes was significantly different between populations. Eight of the polymorphisms involved six transporter genes: the *ABCB1, ABCC2*, *ABCC4*, *ABCG2*, *SLC22A7*, and *SLC29A1* genes. In the case of the phase I genes, there were two groups, the *CYP2A6* and the DPYD genes with six significantly different polymorphisms. We also observed significantly different distributions in six polymorphisms in six other genes involved in the ADME processing of drug: the *ITGB5*, *MTHFR*, *RRM1*, *TP53*, *TYMP* and *UMPS* genes.

By contrast, we identified only 12 polymorphisms of nine genes that varied significantly between the IND and the AMR populations. These included one polymorphism in each of the three transporter genes: *ABCG2*, *SLC22A7* and *SLC29A1*. Five polymorphisms were found in the phase I genes, *CYP2A6* and *DPYD*. Four polymorphisms of four other ADME genes were also significantly different, that is, the *ITGB5*, *MTHFR*, *RRM1* and *UMPS* genes.

In the comparison between the IND and the EAS populations, 16 polymorphisms of 11 genes had a significantly different distribution. These included five polymorphisms of four transporter genes: the *ABCC2*, *ABCG2*, *SLC22A7*, and *SLC29A1* genes. Significant differences were also found in the two phase I genes, *CYP2A6* and *DPYD*. A significant level of variation was also found in a further five polymorphisms, of five genes, the *FPGS*, *ITGB5*, *MTHFR*, *TP53* and *UMPS*.

Pairwise comparisons between the IND and the EUR populations revealed significant differences in 18 polymorphisms in 12 genes. Six of these polymorphisms involved five transporter genes: *ABCC2, ABCC4*, ABCG2, *SLC22A7* and *SLC29A1*. In the phase I genes, significant differences were found in one polymorphism of the *CYP2A6* gene and in four polymorphisms of the *DPYD* gene. The analysis also demonstrated significant differences in seven polymorphisms in five other genes related to the ADME: the *ITGB5*, *MTHFR*, *RRM1*, *TYMP*, and *UMPS* genes.

Finally, significant differences were identified between the IND and SAS populations in 16 polymorphisms present in 10 genes. Five of these polymorphisms were present in four transporter genes: *ABCB1*, ABCG2, *SLC22A7*, and *SLC29A1*. Six of the variants were also found in the phase I genes, CYP2A6 and *DPYD*. The five other markers were identified in four other ADME-related genes, *ITGB5, MTHFR, TP53* and *UMPS*.

## Discussion

Adverse Drug Reactions (ADRs) are one of the principal causes of morbidity in developed countries^19^. High rates of hospitalization due to ADRs are caused, in part, by the standardization of drug doses, which overlooks the variability among patients in factors such as the symptoms of the disease, environmental and genetic characteristics, and ethnicity^20,21^.

Another important factor to be considered during treatment choice is drug efficacy, the ability of a drug to achieve the desired effect^22^. The main challenge to achieve maximum effectiveness of a drug is also the interindividual variability in drug response. Drug mechanisms focused on targets with genetic support would succeed twice as often as those without it, reducing the clinical trial’s phases due to lower rates of failure caused by lack of efficacy during clinical development^23^.

Most pharmacogenetic studies focus on Caucasian populations, which cannot necessarily be extrapolated reliably to the application of PGx in other ethnic groups, such as Amerindian, which may have a unique genetic profile resulting from prolonged geographic isolation and inbreeding^24,25^. There have been relatively few studies of molecular biomarkers in Amerindian populations, highlighted by the fact that they are absent from the 1000 Genomes database, the largest available collection of data on human genetic variability.

This emphasizes the need for the investigation in other ethnic groups, in particular Amerindians, of predictive molecular biomarkers known to play a role in specific treatments or other, as yet undiscovered markers, in order to understand the genetic variability of these populations and to use the knowledge to delineate personalized treatment protocols, in order to improve drug security in Amerindian populations from the Amazon region, as well as in other populations with a high degree of admixture with this ethnic group.

In the present study, we compared the genetic variability of Amerindian populations from the Amazon region with five populations from the 1000 Genomes Project, analyzing 26 genes responsible for the absorption, distribution, metabolization, and excretion of drugs. The F_ST_ indices indicate that the Amerindian and African populations were the most distinct genetically, which is consistent with the history of the world’s human populations, in which the Amerindian and African groups represent the extremes of the evolutionary process^26^.

Still regarding the F_ST_ analysis, the lower values of genetic differences with the Amazonian Amerindians is observed on the East Asian population (F_ST_ value = 0.05831), which is mainly composed of Chinese and Japanese individuals. This outcome corroborates with the “*Bering Strait”* hypothesis, which discusses that the settlement of the Americas and the formation of the first Native Americans are due the migrations of Asians populations that occurred less than 15,000 years ago through a land extension known as the Bering land bridge, which joined Northeast Asia and North America^27^.

Relying up on the F_ST_ analysis concomitant with the pairwise comparisons analysis, the Amerindian population is genetically most similar to the American population, with significant differences being found in only 12 polymorphisms, and an F_ST_ value of 0.06042. The American samples from the 1000 Genomes database includes Colombian (CLM), Puerto Rican (PUR), Peruvian individuals (PEL), and Californian residents of Mexican descent (MXL), who are representative of the different populations of Latin America, which implies a certain degree of genetic similarity with the ancestral populations of South and Central America^28^.

In fact, high levels of interethnic admixture have already been shown in populations from the Americas, and in particular, from Latin America^29^. This genetic admixture originated during the European colonization of the New World, when native Americans came into contact with European immigrants arriving on the continent from 1492 onward, and with African immigrants, from 1502 onward^30^.

Gravel *et al*. (2012) investigated the exome of the Colombian, Mexican and Puerto Rican populations from the 1000 Genomes database and estimated the contributions of African, European, and Native American ancestors to these populations. The findings of the study indicated that Amerindians contributed 12.8% of the PUR exome, 25.6% of that from CLM, and 47.6% from MXL^31^. There are still no studies that evaluate the ancestry of Peruvian samples in the 1000 Genomes database, however, Homburger *et al*. (2015) evaluated 119 individuals from Peru and determined that they had an Amerindian ancestry of 68.3%^32^.

The profile of the genomic ancestry of Latin American countries thus indicates considerable heterogeneity in terms of the contribution of Amerindians, resulting from the varied history of the formation of these populations, and their degree of interethnic admixture. The study of ADME polymorphisms in native American populations is, therefore, fundamentally importance to ensure the optimal implementation of healthcare programs that include genomic information on both Latin and Amerindian populations.

Here, we discuss specifically the data on three of the genes – *ABCB1*, *CYP2A6* and *DPYD*– whose profiles in the Amerindian population were significantly different from the other study populations. The impact on the enzyme activity caused by the allelic variants analysed is described at Supplementary Table 1 (Table SI). These genes are widely cited as pharmacogenetic biomarkers capable of significant alterations in the ADME patterns of a number of different classes of drugs, with their clinical significance being recognized in the pharmacogenetic labelling of a number of different medications by prominent drug regulatory agencies, including the American Food and Drug Administration (FDA), the European Medicines Agency (EMA), the Japanese Pharmaceuticals and Medical Devices Agency (PMDA), and the Canadian Health Canada Santé Canada (HCSC)^33^.

### *ABCB1* and Aliskiren

The *ABCB1* gene (P-glycoprotein encoding [P-gp]) belongs to the ATP-binding cassete (ABC) superfamily of human carriers, and plays a key role in the absorption, distribution, and elimination of drugs within the organism, being considered one of the major determinants of drug resistance^34^. The rs1128503 and rs1045642 polymorphisms of the *ABCB1* gene have been identified as have a potential role in the response to and toxicity of a number of different drugs, including imatinib, opioids and treatments for epilepsy^35–37^.

The European and Canadian drug regulatory authorities require the inclusion of pharmacokinetic information on their drug labels on the interaction between the *ABCB1* gene and Aliskiren (Rasilez HCT^®^), an antihypertensive drug. The EMA identifies *ABCB1* as the principal efflux system involved in the intestinal absorption and biliary excretion of the drug and contraindicates the concomitant administration of Aliskiren and medications that may influence *ABCB1* gene action^38^. The HCSC also refers to the role of the *ABCB1* gene in the Aliskiren efflux system in its drug labelling, contraindicating the use of different glycoprotein inhibitors and other drugs during treatment with Aliskiren^39^. Polymorphisms on the *ABCB1* gene also may cause a polymorphic enzyme of P-gp with diminished activity^40^, therefore, should also be considered as an influence factor of Aliskiren efficacy.

In the present study, we found high allele frequencies of the *ABCB1* variants (of at least 40%) in the Amerindian populations from the Amazon Region. These variants may reduce glycoprotein activity and thus alter the metabolization of antihypertensive drugs, such as Aliskiren, affecting its efficacy in the treatment of Amerindian populations.

### *CYP2A6* and Letrozole

The cluster of *CYP2* genes on chromossome 19 includes six families, one of which is the *CYP2A* family, with three genes, the *CYP2A6, CYP2A7*, and *CYP2A13*. The *CYP2A6* gene is highly polymorphic, and its protein product represents one of the principal liver enzymes responsible for the metabolism of drugs and xenobiotics^41^.

The Japanese Pharmaceutical Regulatory Agency (PMDA) includes pharmacogenetic information on the *CYP2A6* gene on its Letrozole label. Letrozole is a chemotherapeutic drug used to treat breast cancer. *In vitro* studies have shown that it is metabolized primarily in its inactive form in liver microsomes through the action of the *CYP2A6* gene^42^.

Patients identified as poor or normal metabolizers based on their *CYP2A6* polymorphisms, which modify enzyme activity, may suffer either an increased toxicity profile or treatment shortcomings. There is a wide range of functional activity data for the described variants of *CYP2A6*, leading to either differences in enzyme activity or mRNA expression and, consequently, protein levels^43^. Patients with specific polymorphic variants or a combination of *CYP2A6* polymorphisms that define them as poor metabolizers may present a twofold increase in their plasmatic concentrations of Letrozole when compared with extensive metabolizers^42^.

One of the markers associated with the poor metabolizer profile is CYP2A6*9 (rs28399433), which was extremely frequent (46%) in the Amerindian population, and much higher than in the other populations, in which the mean frequency is 13%. This indicates that the Amerindian population from the Amazon region has an abnormally high proportion of poor metabolizers, due to the frequency of the CYP2A6*9 variant, which may determine adverse toxic reactions during treatment with Letrozole, and other drugs that are metabolized by the *CYP2A* subfamily.

### *DPYD* and Capecitabine + Fluorouracil

The *DPYD* gene is composed of 23 exons at the 1p22 chromosomal locus. This gene encodes the enzyme dihydropyrimidine dehydrogenase (DPD), which accounts for about 85% of the hepatic catabolism of 5-fluorouracil to its inactive metabolic form, 5- fluoro-5,6-dihydrofluorouracil^44^. Because this is an essential enzyme in the metabolism of 5-fluorouracil, patients with low DPD enzyme activity have a higher risk of developing severe and even lethal toxicities during standard chemotherapeutic treatment with this drug^45^.

The guidelines of the Clinical Pharmacogenetics Implementation Consortium (CPIC) indicate three *DPYD* genotypes as the major non-functional variants of this gene: *2 A (rs3918290), *13 (rs55886062), and rs67376798. The CPIC guidelines characterize phenotypes based on each of these three variants, and strongly recommend the use of alternative drugs or the reduction of the standard 5-Fluorouracil dose by 50% for patients who are either homozygous or heterozygous for any of these three variants^46^.

A number of national drug regulatory authorities include information on the *DPYD* gene in their pharmacogenetic drug labels. Capecitabine is one of the major 5- Fluorouracil prodrugs used to treatment various types of solid neoplasias. The FDA includes a warning note in its label for this drug (XELODA^®^), advising patients who have genotypes associated with an absence or reduced activity of DPD that they are at a higher risk of developing severe or potentially lethal adverse reactions^47^.

The FDA also includes pharmacogenetic information labels on the potential for severe toxicity in patients with DPD deficiency during treatment with Fluorouracil, describing the different types of adverse reaction presented by patients with homozygous or heterozygous mutations resulting from a partial or total deficiency of the DPD enzyme. The FDA recommends discontinuing permanently the use of Fluorouracil based on the severity of the toxicities observed in patients treated by the 5- FU regime^48,49^.

Nine polymorphic variants of the *DPYD* gene were identified in our Amerindian population. The allele frequencies of the rs17116806, rs1801159 and rs4970722 markers were all higher than 40%, contrasting with the much lower frequencies found in all other populations around the world. Two of three deleterious polymorphisms mentioned in the CPIC guidelines were identified in our Amerindian population. The frequency of the rs3918290 marker was 1%, similar to that of most other populations, in particular those from Asia. The rs55886062 marker had a frequency of 2%, however, which is relatively high in comparison with the other populations, in which it did not exceed 1%, when present.

In our Amerindian population, the frequency of the rs1801133 mutation was consistent with that found in the other study populations (mean = 23%), whereas the rs1801131 polymorphism had a low frequency (9%) compared to the other populations (mean = 25%). This indicates a lower risk of developing serious adverse effects in Amerindian women, associated with the use of contraceptives based on norelgestromin and ethinylestradiol.

Up until now, there has been no systematic assessment of the potential response of Amerindian ethnic groups from the Amazon basin to treatment with drugs known to be influenced by genetic polymorphisms. Our study evaluated the unique features in the genetic profile of 26 ADME-related genes in an Amazonian Amerindian population comparing with others continental populations, which may reflect a distinct therapeutic profile in relation to the efficacy and toxicity of drugs marketed widely for the treatment of a range of diseases. Given this, there is a clear need for the understanding of the individual genetic profiles of Amerindians, and the application of this knowledge to the design of specific pharmacogenomic-guided treatment protocols for indigenous Amazon ethnic groups. This approach would also be valid for populations, such as that of Brazil, which present a high degree of miscegenation with this indigenous group. As future perspectives of our research group regarding this study, data on other clinically better known ADME polymorphisms will further be reported in a separate paper.

## Supplementary information

Table SI. Functional Activity of the allelic variants of ABCB1, CYP2A6 and DPYD gene.
