# Supplementary material for: Polymorphisms of ADME-related genes and their implications for drug safety and efficacy in Amazonian Amerindians
Source: Sci Rep. 2019 May 10;9:7201. doi: 10.1038/s41598-019-43610-y (PMC6510895; doi:10.1038/s41598-019-43610-y)
Supplement: Supplementary file 1 — Table SI. Functional Activity of the allelic variants of ABCB1, CYP2A6 and DPYD gene. [file 41598_2019_43610_MOESM1_ESM.pdf]

Polymorphisms of ADME-related genes and their implications for drug safety and efficacy in Amazonian Amerindians

Juliana Carla Gomes Rodrigues[1,4], Marianne Rodrigues Fernandes[1,4], João Farias Guerreiro[2], Artur Luiz da Costa da Silva[3], Ândrea Ribeiro dos Santos[1,2], Sidney Santos[1,2], Ney Pereira Carneiro dos Santos*[1,2].

[1]*Research Center of Oncology, João de Barros Barreto University Hospital, Federal University of Pará, Belém, Brazil*
[2]*Laboratory of Human and Medical Genetics, Institute of Biological Science, Federal University of Pará, Belém, Brazil.*
[3]*Genomics and Bioinformatics Laboratory, Institute of Biological Science, Federal University of Pará, Belém, Brazil.*
[4]*These authors contributed equally as first authors.*

Table SI. Functional Activity of the allelic variants of *ABCB1*, *CYP2A6* and *DPYD* gene.

Gene	SNP ID	Alleles	Functional Activity of the enzyme[a]
ABCB1	rs1045642	G>A	Decreased
ABCB1	rs1128503	G>A	Normal
CYP2A6	rs28399433	A>C	Decreased
CYP2A6	rs8192726	C>A	Decreased
DPYD	rs17116806	C>A	Normal
DPYD	rs17376848	A>G	Decreased
DPYD	rs1760217	A>G	Increased
DPYD	rs1801159	T>C	Normal
DPYD	rs1801265	A>G	Normal
DPYD	rs3918290	C>T	Decreased
DPYD	rs4970722	T>A	Normal
DPYD	rs55886062	A>C	Decreased

[a]Based on the Variant Annotation section on the Pharmgkb database: https://www.pharmgkb.org/
